# Contribution of the Avon Longitudinal Study of Parents and Children to nutritional epidemiology

**DOI:** 10.1093/nutrit/nuv057

**Published:** 2015-09-22

**Authors:** Alison M. Stephen

**Affiliations:** *A.M. Stephen* is with the Department of Nutritional Sciences, School of Biosciences and Medicine, University of Surrey, Guildford, UK.

**Keywords:** ALSPAC, child development, dietary patterns, epidemiology

In the late 1980s, Professor Jean Golding at the University of Bristol in England conceived the idea of a birth cohort in Bristol and its surrounding area, to investigate the ways whereby genes and the environment interact to affect the health, behavior, and development of children. It had the novel features of recruitment prior to birth of the cohort members and collection of all available biological samples at frequent intervals, referred to as the “collect all” approach. Titled the “Avon Longitudinal Study of Parents and Children” or ALSPAC, the study began recruitment in 1991 of all willing pregnant women as early in pregnancy as possible in Bristol and surrounding health districts. By 1992, there were more than 14 000 pregnant women in the study.

From the outset, it was recognized that dietary intake and the nutritional status of both the mother and the infant might be important considerations for many of the health conditions that could be examined in the study. Dietary assessment was, therefore, included at many of the time points. The first of these was in pregnancy, followed by a number of assessments during infancy and childhood, the last being at the age of 13 years. There is a wide range of dietary assessment methods which could be used in a study of this type and these vary widely in the extent of the detail obtained, but also in the cost of producing the final data from the food intakes recorded. The methods used in the ALSPAC at the various time points were those that were feasible for the numbers and group being studied and the level of funding available. Unlike some studies, the dietary data in the ALSPAC has been used for analyses for a multitude of conditions and published widely; the first dietary papers appeared in 1997. For all the dietary work, Dr Pauline Emmett has been at the core of the analysis, providing expert nutritional advice to collaborating researchers from many disciplines, from epidemiology to cell biology, from allergy to endocrinology to psychiatry. The involvement of a nutritionist in analyses involving the dietary data has maintained the fundamental principles of nutrition in terms of quantities consumed in relation to recommendations, understanding factors influencing food consumption, placing the findings in the context of typical consumption in western countries, and always relating intakes in the ALSPAC to those from UK national surveys to ensure that results are meaningful for the population at large.

This supplement describes the research findings from the ALSPAC in relation to dietary intake assessed in pregnancy and childhood. (A previous publication summarized the results for diet in infancy.) Dr Emmett has been the main author of these reviews. The reviews summarize findings from the numerous publications that have appeared in the literature in the last two decades, and it is to those original papers that one should go for more detailed information and supporting literature. The reviews presented here provide the overall findings of the research and place them in the context of current knowledge about each of the topics covered.

The first review article is on diet in pregnancy, its relationship to some biological measures in the mothers and effects on health outcomes in the offspring that may be influenced by the quantity and quality of the maternal diet. In this case, the diet was assessed using a food frequency questionnaire (FFQ). This is an appropriate method for the number of individuals assessed, although it lacks the detail that more complete methods provide. For some foods, it is not the best method for investigation, but for infrequently consumed foods, it is a better method than records over a few days or a recall covering only 1 or 2 days. One of the foods of particular interest in pregnancy is fish, because of its content of omega-3 fatty acids and iodine and the role these may play in child development, particularly cognitive development and visual acuity. An area of focus in the ALSPAC has been examining the role that fish and omega-3 fatty acids may play in the maternal diet and there are a sizeable number of reports on this topic in the present review article. These have shown associations between fish intake and maternal depression and anxiety as well as associations with neurocognitive development in the offspring, findings that were also demonstrated for omega-3 fatty acids in maternal blood and iodine from urine samples.

The second review article in the present supplement is concerned with the children in the ALSPAC cohort themselves; it describes publications and results of studies on dietary intakes, growth, and measures of and the development of obesity. The multiple assessments performed in the ALSPAC enable the cohort to be useful for assessing many measurements themselves. For example, it has been used to assess the reference standards for obesity and how the cohort would be differently described using the various cut-offs for overweight and obesity that have been suggested. With objective measures of weight, fat mass, and physical activity, the assumptions generally used to assess under-reporting of dietary intake could be put to the test and compared and the usefulness of waist circumference as a measure of fat mass has been compared to fat mass assessed using dual-energy X-ray absorptiometry (DXA) measurements. Although not intended as a major purpose of the cohort, the size of the cohort and the repeated detailed measurements have allowed such investigations, enabling the best methods to be used in other studies without such detailed information and providing estimates of the accuracy and validity of such measurements.

This paper also describes trends in consumption by the children, how dietary intakes changed as they grew, and how well their intakes compared to recommended intakes at different time points in childhood. Comparisons were frequently made to the National Diet and Nutrition Surveys (NDNS) in the UK, which are representative cross-sectional surveys of dietary intakes of the population. The similarities between NDNS surveys conducted at similar time points and the ALSPAC enable determination of how representative of the country the cohort has been; more recent NDNS surveys enable comparison over time to see how current food and nutrient intakes differ from those in the 1990s when the ALSPAC cohort were children; comparisons have also been made to a cross-sectional survey conducted in the UK in 1987, allowing estimates of changes from that time to the 1990s when the ALSPAC cohort was measured. Taking these time variations into account enables the ALSPAC’s usefulness to be extended beyond the time in which it was conducted.

The cohort is one of the few that allows the diets of children at various ages to be compared with the diets of their parents, and in the ALSPAC, the children’s diets were found to closely reflect those of their mothers. This is important if improvements in diet are to be made; in order to change the diets of children for the better, it is necessary to work to improve the diets of their mothers as well. The quality of the children’s diets was also found to be related to the extent of education of their mothers, with better-quality diets associated with greater maternal education. Targeting mothers with less education would seem to be a necessary strategy to improve the diets of the population’s children.

With its many repeated measurements, the ALSPAC has the ability to address popular topics, and obesity and its causation remain a focus of interest. In the ALSPAC, energy density at age 7 years was found to be predictive of higher BMI at age 9 years, with the same finding for diet at age 11 years and BMI at age 13 years. However, contrary to other work and common perception, there was no evidence of a relationship between consumption of sugar-sweetened beverages at age 5 or 7 years and fat mass at 9 years. The versatility of the information available for research in the ALSPAC is apparent with the results of this review.

The third and last article in the supplement focuses on dietary patterns and health. In the last few years, a new approach to investigating dietary intake has become popular, that of dietary patterns. After many decades of investigation of single foods or nutrients in relation to disease, this new approach recognizes that single foods and nutrients are not eaten in isolation, but alongside other foods and nutrients and that there tend to be clusters of behavior in terms of the types of foods eaten together. There are several approaches to the investigation of dietary patterns: principal components analysis (PCA), cluster analysis, and reduced rank regression. The ALSPAC has been at the forefront in the utilization of the dietary patterns approach and was the first longitudinal study to investigate these children. All three methods have been used. These are described in this review and the findings from the work on dietary patterns are discussed. The researchers found that certain patterns are more common in certain groups; for example, a “health conscious” pattern, with higher intakes of whole-grain breads and cereals, fish, and fruit juice is more common among mothers of higher socioeconomic standing, education, and older age, and it is less common in those who were overweight before pregnancy. Patterns have been found to be different in boys compared to girls and they have also been found to track through childhood, as shown by carrying out analysis at different ages. The patterns have also been examined in relation to energy and nutrient intakes. With its multiple time points and repeated dietary information, in some cases achieved with more than one method, the ALSPAC dataset has been able to be used to explore methods of dietary pattern analysis and the best ways to carry these out. Patterns have also begun to be related to health outcomes, such as body weight and cognitive development.

For anyone interested in knowing what has been studied and the results of the ALSPAC research in relation to diet, the 3 articles in the present supplement provide a comprehensive summary of the work to date. They provide much needed information about diet and health in children using a longitudinal approach. Too often, longitudinal studies short-change the dietary aspect and assume that adequate information can be obtained with a few questions or a short questionnaire. With the many interests of researchers involved in longitudinal studies, this is understandable in order to contain respondent burden, but diet is complex and in order to obtain enough detail to be able to study it appropriately, considerable emphasis has to be placed on the collection of sufficient information. This has been done in the ALSPAC from pregnancy to age 13 years and the benefits of doing so are clear from these reviews. Sadly, there is no dietary data from later teenage years or early twenties in the ALSPAC dataset; this is largely due to an inability to obtain funding, rather than changing attitudes with regards to the study’s importance. There needs to be greater recognition of the role that diet plays in health and the need for dietary information to be collected in detail in longitudinal studies. The ALSPAC remains the birth cohort with the greatest quantity of dietary data for childhood and this, alongside the wealth of information on other aspects of life and health, continues to make a valuable contribution to nutritional epidemiology. For anyone contemplating a longitudinal study or for those beginning research in this type of work, the reviews in this supplement are an excellent starting point to show what can be done.

